# Characterization of Conjugates between α-Lactalbumin and Benzyl Isothiocyanate—Effects on Molecular Structure and Proteolytic Stability

**DOI:** 10.3390/molecules26206247

**Published:** 2021-10-15

**Authors:** Jenny Spöttel, Johannes Brockelt, Sven Falke, Sascha Rohn

**Affiliations:** 1 Institute of Food Chemistry, Hamburg School of Food Science, University of Hamburg, Grindelallee 117, 20146 Hamburg, Germany; jenny.spoettel@chemie.uni-hamburg.de (J.S.); johannes.brockelt@chemie.uni-hamburg.de (J.B.); 2 Laboratory for Structural Biology of Infection and Inflammation, Institute of Biochemistry and Molecular Biology, University of Hamburg, Notkestr. 85, 22603 Hamburg, Germany; falke@chemie.uni-hamburg.de; 3 Department of Food Chemistry and Analysis, Institute of Food Technology and Food Chemistry, Technische Universität Berlin, TIB 4/3-1, Gustav-Meyer-Allee 25, 13355 Berlin, Germany

**Keywords:** rotein modifications, α-lactalbumin, whey proteins, benzyl isothiocyanate, cd spectroscopy, hydrophobicity, hydrodynamic radius, tryptic digestion, mass spectrometric analysis

## Abstract

In complex foods, bioactive secondary plant metabolites (SPM) can bind to food proteins. Especially when being covalently bound, such modifications can alter the structure and, thus, the functional and biological properties of the proteins. Additionally, the bioactivity of the SPM can be affected as well. Consequently, knowledge of the influence of chemical modifications on these properties is particularly important for food processing, food safety, and nutritional physiology. As a model, the molecular structure of conjugates between the bioactive metabolite benzyl isothiocyanate (BITC, a hydrolysis product of the glucosinolate glucotropaeolin) and the whey protein α-lactalbumin (α-LA) was investigated using circular dichroism spectroscopy, anilino-1-naphthalenesulfonic acid fluorescence, and dynamic light scattering. Free amino groups were determined before and after the BITC conjugation. Finally, mass spectrometric analysis of the BITC-α-LA protein hydrolysates was performed. As a result of the chemical modifications, a change in the secondary structure of α-LA and an increase in surface hydrophobicity and hydrodynamic radii were documented. BITC modification at the ε-amino group of certain lysine side chains inhibited tryptic hydrolysis. Furthermore, two BITC-modified amino acids were identified, located at two lysine side chains (K32 and K113) in the amino acid sequence of α-LA.

## 1. Introduction

It is well known that a balanced diet is crucial for maintaining good health [1]. With the aim of increasing the consumption of vegetables with their health-promoting secondary plant metabolites, different recipes of traditional foods are improved with higher amounts of vegetables. Especially, vegetables of the plant order Brassicales are associated with multiple health-promoting properties, largely due to the glucosinolates (GLS) or their hydrolysis products, in particular, isothiocyanates (ITC) [2,3,4,5]. For example, some studies reported anti-inflammatory, antibacterial, as well as antidiabetogenic effects, and it has even been shown that consumption of vegetables rich in GLS can reduce the risk of developing certain types of cancer [6,7,8,9,10,11,12,13,14,15,16].

**Figure 1:**
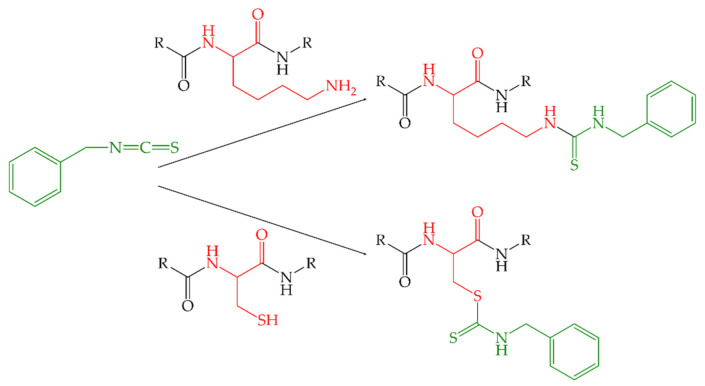
Reaction of benzyl isothiocyanate (BITC) with nucleophilic groups, e.g., of protein side chains. Top: reaction with an amino group to form a thiourea. Bottom: reaction with a thiol group to form a dithiocarbamate, redrawn from [17].

Due to their high electrophilicity, ITC can interact with proteins in a diverse bunch of foods. They react with nucleophilic groups such as thiol and amino groups in proteins to form thiocarbamates and thioureas (Figure 1), and thus have a significant influence on various protein properties [5].

In addition to the formation of reaction products in complex food matrices during food production and processing, interactions between proteins and ITC in the human organism are also conceivable [18,19,20]. In most cases, such reactions are often considered negatively because they can reduce the bioavailability of the health-promoting secondary metabolites and essential amino acids, as well as affect the physicochemical properties and functionality of proteins [6,9]. However, there might also be a possibility of using the knowledge on the interactions as an opportunity to selectively alter the properties and functionality of proteins [1,7,9,21,22,23].

**Figure 2:**
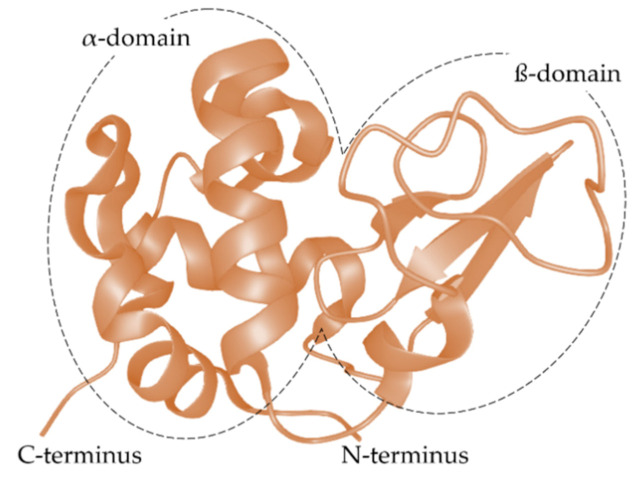
Ribbon model of the conformational structure of bovine α-lactalbumin (PDB ID 1F66S), redrawn from [37].

Recent research is focusing more and more on the interactions between different food compounds/ingredients. Besides being a good model for studying protein alterations, whey proteins are also very important food proteins. They are of particular interest due to their high content of branched, sulfur-containing, and further essential amino acids. In addition to their outstanding nutritional quality, their functional properties (e.g., gelling, emulsifying, foaming) make them valuable ingredients in different foods/recipes. Furthermore, they have good solubility in a wide pH range and can serve as carriers for bioactive substances, as their structure is characterized by different domains and hydrophobic pockets [1,24,25,26,27]. Next to β-lactoglobulin (β-LG), α-lactalbumin (α-LA) is the second most abundant whey protein in cow’s milk [24]. α-LA is an acidic, monomeric protein with a molecular weight of 14.2 kDa, whose amino acids are well characterized, and its sequence is composed of 123 amino acids [28,29,30,31,32,33,34,35]. The structure of α-LA is further characterized by four disulfide bonds involving all eight cysteines and a calcium-binding site, which ensures the correct folding and high molecular stability of α-LA [24,36,37,38,39]. While the secondary structure of native α-LA consists of 26% α-helices, 14% β-sheets, and 60% disordered structures [36], the tertiary structure is built up from two domains: a large α-domain and a small β-domain (Figure 2) [37].

Previous studies showed that the interaction of β-LG with secondary plant metabolites can mask the bitter taste of the latter and contribute to its stabilization and solubility [40,41]. In addition, it was found that the covalent modification with ITC affected the physiological and technofunctional properties of proteins. For example, it was documented that the binding of ITC to several proteins resulted in a change in protein conformation [9,42,43,44]. Further studies proved that the binding of allyl isothiocyanate (AITC, a hydrolysis product of the glucosinolate sinigrin) to β-LG resulted in a change in the folding and structure of the protein accompanied by an optimization of technofunctional properties such as heat aggregation, foam formation, and emulsification compared to untreated protein [1]. Derivatization of AITC to whey protein isolate also showed an effect on secondary structure depending on pH value. This was accompanied by changes in physicochemical properties such as aggregation, charge, and hydrophobic regions of the protein surface [9]. In addition, it was reported that the binding of the hydrophilic lysine side chains with hydrophobic ligands, such as those represented by the ITC, resulted in a decrease in water solubility and isoelectric point [45,46]. Similarly, Rawel et al. (2002), who studied the interaction of ITC with various plant proteins, showed that the derivatives exhibited a decreased solubility and a reduced number of free ε-amino groups, which was accompanied by increased hydrophobicity. In addition, inhibition of tryptic degradation of the ITC derivatives was documented as well [42]. It was reported that, depending on the amount of AITC used, steric blocking of the tryptic cleavage sites of β-LG was caused, resulting in elongated peptides and affecting the amount of bioactive peptides after proteolytic hydrolysis [7].

In a recent publication, the allergenic properties of unmodified and BITC-modified α-LA and their hydrolysates have already been estimated [47]. A significant influence on the allergenicity of the protein conjugates, but also on the residual antigenicity of the peptides after tryptic digestion, was found. It was hypothesized that as a result of the BITC-addition, there was a change in protein conformation and a corresponding influence on allergenic properties [47]. However, this needs confirmation, as the effects of covalent interactions are complex and can influence protein folding or the three-dimensional structure successively, being crucial for technofunctional and functional properties (e.g., viscosity, gelling, foam formation, solubility, emulsification, color, smell, and taste), as well as biological characteristics such as digestibility [48,49,50]. Although the reactions of ITC and whey proteins have already been studied several times, it is not yet possible to predict the effect of modification on protein structure and function [1,51]. Further structural characterization of unmodified and ITC-modified proteins remains highly important to estimate the impact on protein structure and functionality and to avoid or even use such covalent modifications to optimize protein functionality in the future.

Based on those findings, the present study aimed at characterizing the influence of the binding benzyl isothiocyanate (BITC), which is a degradation product of the glucosinolate glucotropaeolin to the whey protein α-LA as a model. It was hypothesized that BITC-conjugation affects the structure and conformation of α-LA and, consequently, its properties and functions. The molecular and supramolecular properties of α-LA, as well as digestibility by trypsin upon interaction with BITC, were investigated. For this purpose, an estimation of the secondary structure elements by circular dichroism spectroscopy and an investigation of the surface hydrophobicity using a hydrophobic probe of the BITC-α-LA conjugates were performed. Furthermore, the size of the BITC-adducts and the aggregation behavior were studied using dynamic light scattering. Finally, digestibility with the protease trypsin before and after ITC conjugation was analyzed.

## 2. Results

### 2.1. Determination of Free Amino Groups Using O-Phthaldialdehyde

In the present study, free amino groups in untreated and BITC-modified α-LA were determined. The aim was to characterize the effect of BITC conjugation on the amount of residual free amino groups of α-LA and to describe the degree of modification based on the change in spectrophotometrical absorbance. Table 1 lists the average contents of the free amino groups along with the standard deviation (SD) as a function of the molar ratios used (B_BITC/α-LA_).

The measurement of the α-LA control (B_BITC/α-LA_ = 0) represents the maximum amount of free amino groups in the unmodified protein and sets the basis for the evaluation of residual amounts. To facilitate comparison of the spectra, normalization to the free amino group content of the α-LA control was performed. Table 1 shows a decrease in the amount of free amino groups from 100% to 59.0% with increasing B_BITC/α-LA_ from 0 to 1500. The mean percental amount of free amino groups available in the α-LA derivatives with a B_BITC/α-LA_ molar mass ratio of 10 and 25 decreased by approximately 12–13%. While a further reduction in accessible amino groups to 79.3 ± 2.1% was noted at a molar mass ratio B_BITC/α-LA_ of 50, the results indicated saturation when molar mass ratios B_BITC/α-LA_ were increased further from 500 to 1500. With increasing molar mass ratios B_BITC/α-LA_ from 50 to 1000 or 1500, a further decrease of free amino groups was noted with 61.8 ± 17.5%, 59.9 ± 15.4%, and 59.0 ± 8.8%, respectively.

### 2.2. Investigation of Secondary Structure by Far-UV Circular Dichroism Spectroscopy

**Figure 3:**
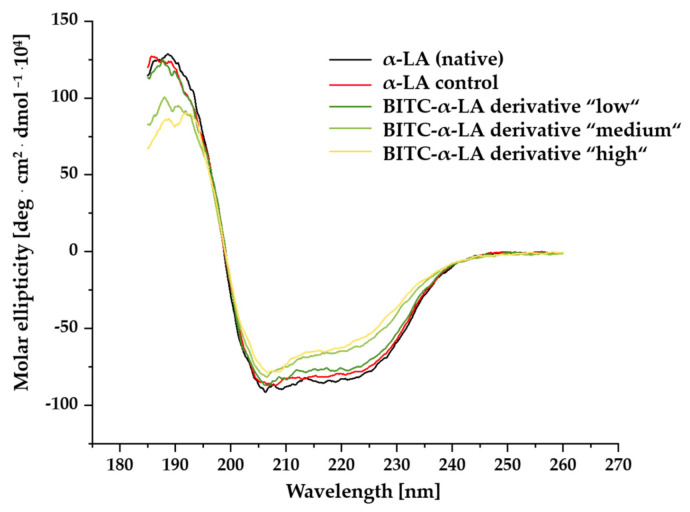
Far-UV CD spectrum of α-LA in the absence and presence of BITC.

For evaluating structural changes of α-LA derivatives, circular dichroism (CD) spectroscopy was performed in the far UV range from 185 to 260 nm to investigate the possible influence of BITC conjugation on the secondary structure of α-LA. Spectra of ellipticity as a function of wavelength were recorded for all samples studied. Figure 3 shows averaged normalized CD spectra obtained for the three BITC-α-LA derivatives, “low”, “medium”, and “high”; the untreated control sample; and the α-LA in the native state.

The CD spectrum of the native α-LA showed a positive maximum at 190 nm and two negative bands at approximately 208 and 220 nm. The CD spectra of the BITC protein derivatives possess a similar shape with minor changes that became more concise with increasing concentrations of BITC used for treatment. While the values of the molar ellipticity of the positive band at approximately 190 nm and those of the negative bands at 208 and 220 nm showed no significant changes between the native, the untreated control sample, and the BITC-α-LA derivative “low,” a decrease in the positive molar ellipticity at 190 nm and an increase in the negative molar ellipticity of the minima at 208 and 220 nm was noted with increasing BITC concentration in the BITC-α-LA derivatives “medium” and “high”.

To gain a better understanding of the structural effects of BITC conjugation, the CD spectra of all samples were subjected to quantitative measurement to evaluate the secondary structure and/or to estimate the changes caused in the secondary structure. Quantitative determination of the percentages of secondary structures of three BITC-α-LA derivatives “low”, “medium” and “high”; the untreated control sample; and the native α-LA was performed using the K2d method in DichroWeb (http://dichroweb.cryst.bbk.ac.uk/html/links.shtml; accessed on 10 October 2021) and is summarized in Table 2.

The results showed that native α-LA consisted of approximately 30% α-helix, 12% β-sheet, and 58% regions of low structural complexity. Binding of BITC induced variations in the content of α-helix and β-sheet structures. With increasing BITC concentration, the amount of α-helix successively decreased from 29% to 17% and, at the same time, the amount of β-sheet structures increased from 12% to 28%. No significant change due to BITC conjugation was observed for the regions with lower structural complexity.

### 2.3. Investigation of Temperature-Induced Conformational Changes Using Far-UV CD Spectroscopy

**Figure 4:**
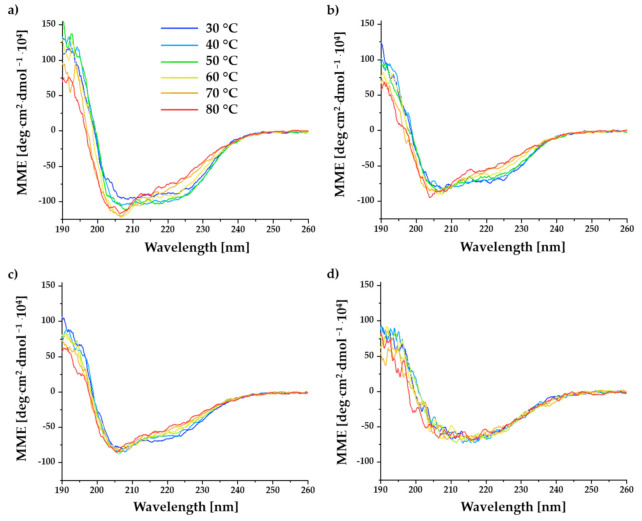
CD spectra of the BITC-α-LA derivatives recorded at different temperatures ranging from 30 to 80 °C. Control sample (**a**), BITC-α-LA derivative “low” (**b**), BITC-α-LA derivative “medium” (**c**), and BITC-α-LA derivative “high” (**d**).

In the following, under the aspect of evaluating the temperature-induced conformational changes of BITC-α-LA derivatives, far-UV CD spectroscopy was performed at different temperatures. Knowledge about the temperature-dependent folding stability of α-LA is of great interest in food technology, as it significantly influences the functional and nutritional properties of foods (e.g., dairy products in the case of α-LA). For this purpose, CD spectra of the untreated and BITC-treated α-LA were recorded in a temperature range from 30 to 80 °C with a heating interval of 2 °C/min. Figure 4 shows the CD spectra of the unmodified α-LA control (a) and the BITC-α-LA derivatives “low” (b), “medium” (c), and “high” (d).

The CD spectrum obtained for the α-LA control (Figure 4a) showed a decrease in the molar ellipticities of the double minimum and an increase in the molar ellipticities of the maximum at 190 nm in the temperature range from 30 to 50 °C. While the molar ellipticity of the minimum at 208 nm continued to decrease with increasing temperature (60–80 °C), the molar ellipticity of the minimum at 222 nm increased. In parallel, a decrease of the maximum at 190 nm was observed in the temperature course from 60 to 80 °C.

In the CD spectra of the BITC-α-LA derivatives “low” and “medium” (Figure 4b,c), a steady decrease in the molar ellipticity of the maximum and an increasing molar ellipticity of the minimum at 222 nm could already be observed for temperatures ≥ 40 °C. A significant difference between these two derivatives was that with increasing temperature (from about 60 °C, analogous to the control) in the derivative “low”, the minimum at 208 nm showed a slight decrease in molar ellipticity, while in the derivative “medium” no significant change was observed. It should be emphasized that the differences with increasing temperature were only slightly lower for the BITC-α-LA derivative “medium”, while no significant changes were documented for the BITC-α-LA derivative “high” when evaluated as a function of temperature. In summary, the differences in the CD spectra as a function of temperature became increasingly smaller with an increasing degree of derivatization/modification. Furthermore, the observations described can be supported by comparing the approximated secondary structure compositions for the different derivatives at the different temperatures applied (Table 2; 20 °C and 80 °C), corresponding to the CD spectra shown in Figure 4.

### 2.4. Investigation of Surface Hydrophobicity Using Anilino-1-Naphthalenesulfonic Acid (ANS) Fluorescence

**Figure 5:**
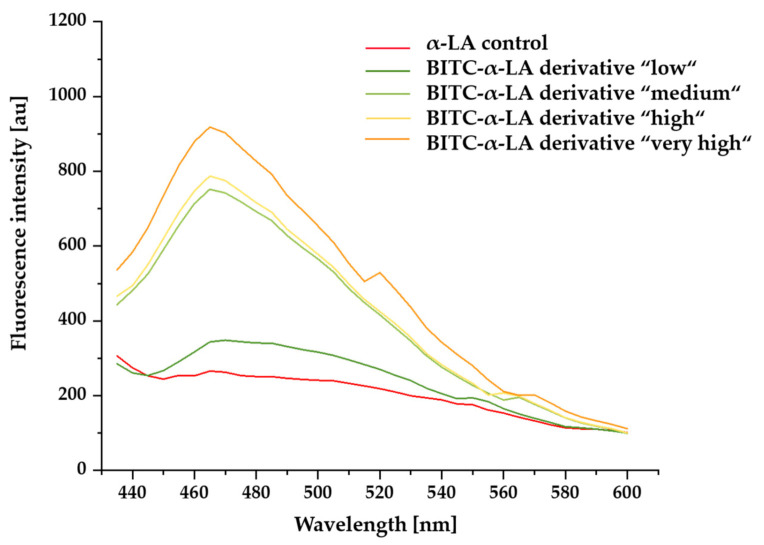
Mean fluorescence intensities versus wavelength of the ANS assay of the BITC-α-LA derivatives.

In the following, the effect of BITC conjugation on the surface hydrophobicity of α-LA was investigated using the extrinsic fluorescent dye anilino-1-naphthalenesulfonic acid (ANS). It can be used to evaluate conformational changes in proteins and to characterize hydrophobic binding sites on the surface of proteins [1,52,53,54,55,56,57,58,59,60]. In Figure 5, the mean ANS fluorescence intensities of the untreated α-LA control sample and BITC-α-LA derivatives “low”, “medium”, “high”, and “very high” are plotted against wavelength.

Low fluorescence emission was observed in the presence of unmodified α-LA (Figure 5, red curve). Depending on the degree of BITC modification, a difference in fluorescence intensity between the measured samples was documented. While the control sample showed a low fluorescence intensity compared to the BITC-treated protein derivatives, a significant increase in ANS fluorescence and a shift of the fluorescence maximum was observed with an increasing degree of modification. Measurement of the ANS fluorescence of the BITC-α-LA derivative “low” showed only a slight increase in intensity and hardly any effect on the position of the fluorescence maximum (Figure 5, dark green curve). With an increasing degree of modification of the α-LA derivatives, the ANS quantum yield could be increased up to a threefold value, and a blue shift of the fluorescence maximum was observed.

### 2.5. Determination of the Hydrodynamic Radius Using Dynamic Light Scattering

**Figure 6:**
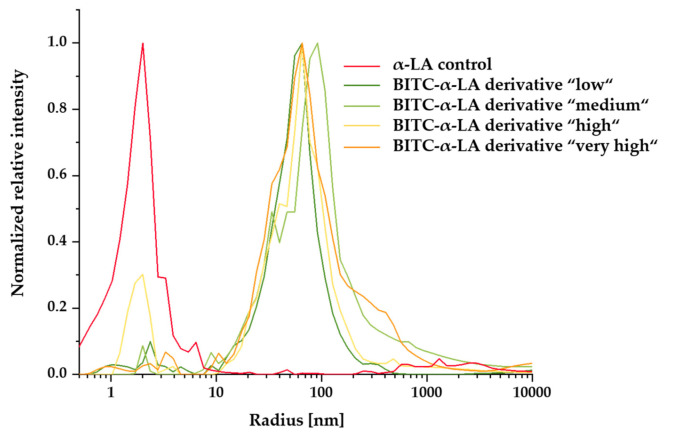
Distribution of hydrodynamic radii of the α-LA control and the BITC-α-LA derivatives.

Using dynamic light scattering, the hydrodynamic radius and aggregation behavior of the untreated protein and BITC-α-LA- derivatives were evaluated. Figure 6 shows the size distribution histograms of the untreated α-LA protein control and the BITC-α-LA derivatives “low”, “medium”, “high”, and “very high”. For each sample, 20 scans were acquired, resulting in the average hydrodynamic radius and the correspondingly standard deviation (Table 3).

For the untreated α-LA control, the hydrodynamic radius was 1.8 ± 0.2 nm, indicative of monomeric α-LA. Moreover, only a single signal with a small peak width was observed in the histogram of the unmodified α-LA control, leading to the assumption that the solution was monodisperse and homogeneous. In contrast, multiple particle sizes and broader peaks were observed for the BITC-α-LA derivatives. Depending on the degree of modification, increased hydrodynamic radii from 13.8 to 89.4 nm were observed, indicating a polydisperse and inhomogeneous solution of large multimers.

### 2.6. LC-ESI-MS/MS Analysis of Unmodified and BITC-Modified α-LA Hydrolysates

**Figure 7:**
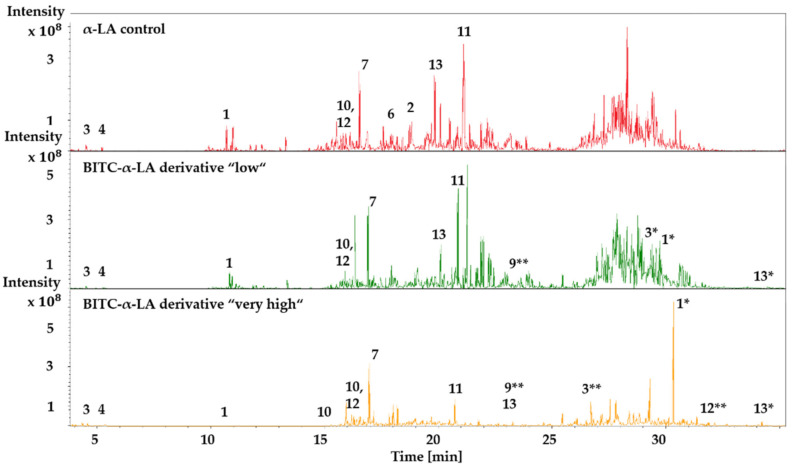
Total ion chromatograms (TIC) of the tryptically hydrolyzed untreated α-LA (red) and the tryptically hydrolyzed BITC-α-LA derivatives “low” (green) and “very high” (orange). The main peptide signals were labeled numerically. Identical peptide sequences are marked with the same number, corresponding peptides that have been modified are marked with an “*”, while elongated peptides resulting from BITC modification were highlighted with “**”. The numbering of the peptides is given in Table 4.

One aim of these approaches was to identify the side chains of the α-LA protein modified with BITC and to investigate the influence on the digestibility of the protein derivatives. For this purpose, the four different BITC-α-LA derivatives, “low”, “medium”, “high” and “very high”, with different BITC concentrations and the unmodified α-LA control sample were subjected to tryptic digestion. The protein hydrolysates were then analyzed by LC-MS/MS. The comparison of the total ion chromatogram of the α-LA control and the BITC-α-LA derivatives “low” and “very high” is shown exemplarily in Figure 7.

Upon reaction of α-LA with BITC and subsequent hydrolysis to peptides, new signals representing BITC-modified peptides were observed. A corresponding decrease in signal intensity of the unmodified peptides was noted as well. Overall, it was found that a mixture of modified and unmodified peptides was the result for the BITC derivatives. The elution of the protein hydrolysates took place between 5 and 35 min.

The *PeptideMass* tool at www.expasy.org (accessed on 20 September 2021) was used to run an in silico tryptic hydrolysis of the α-LA protein. Here, the theoretically generated peptides, as well as their mass and positions in the protein sequence, were provided. The protein sequence of α-LA was taken from the UniProtKB database (http://www.uniprot.org/; accessed on 20 September 2021); under UniProt entry name LALBA_BOVIN and file number P00711). Theoretically, tryptic hydrolysis of native, unmodified α-LA generates 14 peptides. Table 4 lists all identified amino acid sequences of the control sample as well as the derivatives.

Of the 14 theoretical peptides, 10 could be detected in the α-LA control, whereas 8 unmodified peptides could still be identified in all BITC-α-LA derivatives. The peptide sequences IWCK and CEVFR could only be observed for the control. In addition to the eight unmodified peptides of the derivatives, several modified peptides were detected. A total of 11 peptides were identified for the BITC-α-LA derivative “low”, 12 peptides for the BITC-α-LA derivative “medium,” 13 peptides for the BITC-α-LA derivative “high,” and 15 peptides for the BITC-α-LA derivative “very high”. Compared to the native protein, a higher number of peptides were identified after BITC conjugation because of the total sum of unmodified and modified peptides. Furthermore, a concentration-dependent modification of the peptides was observed (Table 4). While the modified *N*-terminal and *C*-terminal sequences EQLTK and LDQWLCEK, respectively, were identified in all derivatives, other peptides were only derivatized at higher BITC concentrations. For example, the modification of the peptide sequence ILDK could only be identified in the derivatives “high” and “very high” and the sequence ELK only in the derivative “very high”. With an increasing degree of modification, longer peptide sequences were detected. These peptides were ELKDLK (No. 3**), KILDK (No. 9**), and ALCSEKLDQWLCEK (No. 12**). BITC-modified ELKDLK was detected in the derivatives “medium”, “high”, and “very high”, whereas BITC-modified KILDK was identified only in the derivatives “high” and “very high”. The modified peptide sequence ALCSEKLDQWLCEK was only observed in the derivative “very high”. Furthermore, as obvious from Figure 6 and Table 4, BITC-modified peptides were eluted at later retention times. For example, the unmodified peptide EQLTK was eluted at 10–11 min in the α-LA control and derivatives, whereas the corresponding modified peptide was eluted at 30 min. Similar effects have been observed for the peptides ELK, ILDK, and LDQLCEK.

#### Identification of BITC-Modified Amino Acids

**Figure 8:**
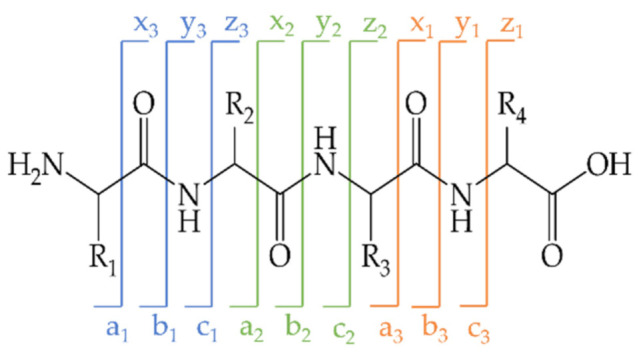
Designation of *N*- and *C*-terminal peptide fragments; redrawn from [62,63].

In mass spectrometry, information about the amino acid sequence of peptides/proteins can be obtained using different fragmentation methods [61]. On the one hand, it is possible to reconstruct the amino acid sequence of peptides and identify proteins from the fragmentation patterns. On the other hand, post-translational modifications such as BITC modification can be identified and localized as well. Depending on the fragmentation method chosen, different breaking points in the backbone of the peptides can be observed. For example, fragmentation yields *a*- and *x*-ions before peptide binding, *b*- and *y*-ions in peptide binding, and *c*- and *z*-ions following peptide binding. While the fragments of the *C*-terminal series are defined as *x*-, *y*-, and *z*-ions, the fragments of the *N*-terminal series are designated as a, b, and c (Figure 8). The labile amide bond in peptides is particularly susceptible to fragmentation, resulting in a breakage of the backbone between individual amino acids and resulting in *N*-terminal *b*-ion series and *C*-terminal *y*-ion series. Peptide bond fragmentation produces a ladder-like representation of the amino acid sequence in the MS/MS spectrum [62,63].

**Figure 9:**
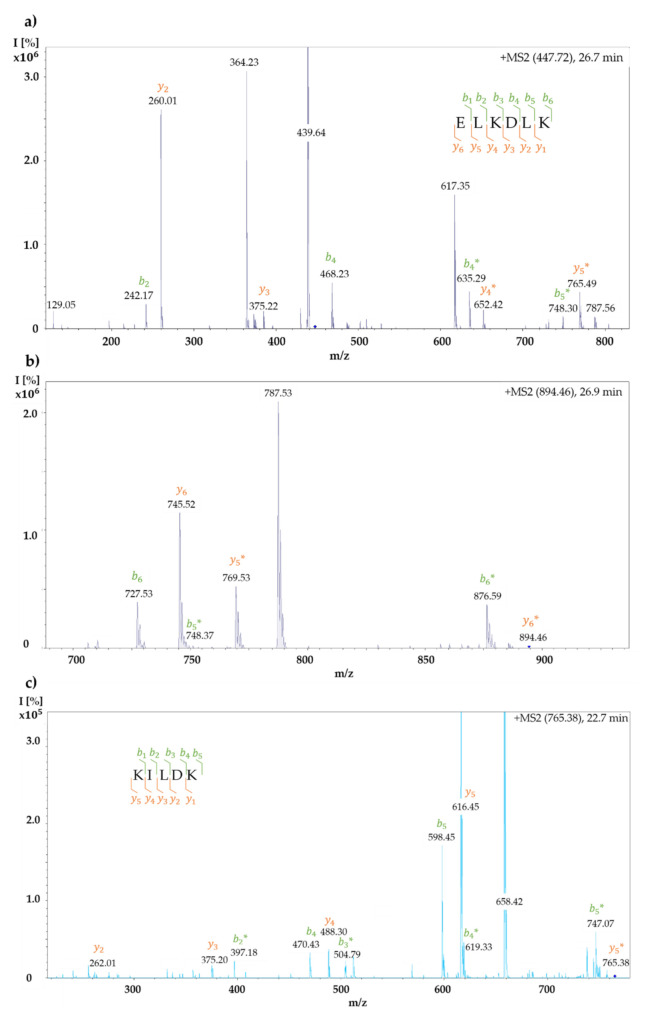
MS/MS spectra of the singly modified peptides ELKDLK of [M + 2H]^2+^ with *m*/*z* 447.72 (**a**) and of [M + H]^+^ with *m*/*z* 894.46 (**b**) and KILDK of [M + H]^+^ with *m*/*z* 765.38 (**c**). The *b*-fragment ions are shown in green and the *y*-fragment ions are shown in orange.

In the present study, a modified amino acid could be identified in the peptide sequences ELKDLK and KILK. As an example, the results are presented using the MS/MS spectra of the derivative “very high”. To identify the modified amino acid, the *y*- and *b*-fragment ions for the corresponding peptides were first calculated using the proteomic tool of the Institute for Systems Biology, Seattle, WA, USA (http://db.systemsbiology.net:8080/proteomicsToolkit/FragIonServlet.html; accessed on 20 September 2021). As posttranslational modifications with BITC (M = 149.21 g/mol) change the mass of an amino acid or peptide, the masses for all *y*- and *b*-ions were calculated in the case of modification at the corresponding amino acid (marked with *). Table 5 and Table 6 show the numbering of the *y*- and *b*-fragment ions and their masses in the native or BITC-modified state (marked with *) for the sequences ELKDLK and KILDK; the identified *y*- and *b*-fragment ions of the modified peptides were illustrated in bold font. Figure 9a,b show the MS/MS spectra of the double- and single-charged ions with an *m*/*z* of 447.72 and an *m*/*z* of 894.46, which can be assigned to the modified peptide ELKDLK. Figure 9c shows the MS/MS spectrum of the modified peptide KILDK with an *m*/*z* of 765.38. The identified *y*- and *b*-fragment ions of the modified peptides were highlighted in orange and green in Figure 9 according to the nomenclature from the Table 5 and Table 6.

The signals in the mass spectrum of the modified peptide ELKDLK could be assigned to the theoretically calculated *y*- and *b*-ions. A nearly complete *y*- and *b*-ion series was observed, enabling the amino acid sequence of peptide ELKDLK to be identified at a retention time between 26.7 and 26.9 min (Figure 9a,b, Table 5). Similary, the *y*- and *b*-fragment ions identified the modified peptide KILDK at a retention time of 22.7 min (Figure 9c, Table 6).

The chemical modification with BITC resulted in a change in the mass of the amino acid or peptide. Thus, the modified amino acid could be identified by corresponding deviating masses of the *y*- and *b*-fragment ions. In the mass spectrum of the modified peptide ELKDLK, the *b6-*, *b5-*, and *b4-* as well as the *y6-*, *y5-*, and *y4*-fragment ions could be detected with the additional mass of BITC. The *b3*- and *b2*, as well as the *y*3 and *y*2-fragment ions, had the mass of the unmodified state, leading to the conclusion that the modification with BITC was present at the central lysine in the amino acid sequence of ELKDLK (Figure 9a,b, Table 5). The mass differences of the *y*- and *b*-fragment ions of the modified peptide KILDK indicated that the BITC-modification occurred at the first lysine of the peptide sequence (Figure 9c, Table 6). However, for the remaining modified peptides, whose masses and sequence could be identified, no clear identification of the modified amino acid was possible due to insufficient detection of the modified *y*- and *b*-fragment ions. Peptides that have been modified are marked with an “*”.

## 3. Discussion

### 3.1. Determination of Free Amino Groups Using O-Phthaldialdehyde

Due to the electrophilic carbon atom of an ITC, a reaction with nucleophiles, such as hydroxy, amino, and thiol groups of protein side chains, is conceivable. While the reactions with thiol groups yield reversible reaction products (dithiocarbamates), the products from the reaction with amino groups are irreversible (thioureas) [17]. Therefore, it was assumed that with increasing BITC concentration (increasing molar ratio B_BITC/α-LA_), an increasing degree of modification of the freely accessible amino groups was achieved, making them unavailable for the reaction with *ortho*-phthaldialdehyde (OPA) and thus, leading to a decrease in absorption.

A total of 13 potential reaction sites for BITC in α-LA were assumed, comprising 12 ε-amino groups of the amino acid lysine (K) and the α-amino group of the *N*-terminal amino acid glutamic acid (E).

The results showed a decreasing trend in the relative mean contents of free amino groups from 100% to 59.0% with increasing concentration of BITC for the derivatization of protein conjugates, indicating the formation of thiourea derivatives and an increasing degree of modification of the protein [1,64,65]. Rade-Kukic et al. (2011) already reported a concentration-dependent reaction between AITC and the whey protein β-LG, observing a decrease in free amino groups with an increasing concentration of AITC present [1]. Similarly, reactions of proteins such as ovomucoid, conalbumin, ovalbumin, myoglobin, insulin, bovine soap albumin, and lysozyme with ITC showed a decrease in the percentage of mean free amino group contents as a function of the amount of ITC used, which was attributed to the formation of thiourea derivatives [43,45,46,66,67,68]. However, a major difference was that those studies used trinitrobenzenesulfonic acid (TNBS) to determine the free amino groups in ITC-protein conjugates. In that alternative method, proteins react with TNBS to form a trinitrobenzene derivative that can be determined photometrically at 416 nm [69]. As some studies concluded that the methods were comparable [70], these results were used for comparison.

Furthermore, a previous study showed that the reaction between AITC and mustard with ITC/protein ratios between 25 and 200 was accompanied by a concentration-dependent decrease in the number of free amino groups from 100% to a maximum of 70%. They described that above an AITC/protein ratio of 100, a constant amount of free amino groups was observed [71]. This result supports the assumption of the present study that there is a stagnant decrease of the free amino groups above a molar mass ratio of 500.

Moreover, a correlation between the decrease in free amino groups and the solubility of ITC-modified bovine sarcoplasma proteins as a function of the ITC concentration used has been reported in the literature [69]. Rawel et al. (1998) showed that the interactions of ITC with various plant proteins affected the physicochemical properties of the proteins by increasing the derivatization of the proteins with ITC. Both solubility and the amount of free ε-amino groups decreased while hydrophobicity increased [42]. Further studies confirmed the correlation between the decrease in free amino groups and the solubility of the derivatives as a function of the amount of ITC used [45,69]. Additionally, the decrease in hydrophobicity of myoglobin as a result of ITC conjugation was confirmed using a hydrophobic fluorescent probe and RP-HPLC [67].

### 3.2. Influence of BITC Conjugation on Secondary Structure of α-LA

Far-UV CD spectroscopy was performed to investigate the effect of BITC conjugation to α-LA on the secondary structure of the protein. Circular dichroism (CD) is the differential absorption of left and right circularly polarized light by optically active (chiral) molecules. Using CD spectroscopy, the wavelength-dependent absorption difference ΔA of left- and right-circularly polarized light is measured, which is defined as dichroism. It is an analytical method that takes advantage of the optical activity of chiral molecules to elucidate the structure of molecules [48,72,73,74]. The reason for the different absorption is that chiral molecules have different refractive indices for right- and left-circularly polarized light [72,74]. In the CD measurements, equal but alternating amounts of left- and right-circularly polarized light of one wavelength pass through an optically active medium. Each light absorption causes a change in the light intensity and consequently in the amplitude of the incident wave. When a chiral substance absorbs left- and right-circularly polarized light to different extents, different amplitudes of the circularly polarized waves result, and the superimposed light is no longer linear but elliptically polarized light [48,72,73,75].

In addition to chiral carbon atoms, optically active chromophores in asymmetric molecular structures such as α-helix, β-sheet can also possess optical activity. Therefore, CD spectroscopy is widely used to analyze the structures of biomolecules such as proteins and DNA [75,76]. In proteins, the peptide bonds are the absorbing group. Their ellipticity changes depending on their conformation. In CD spectroscopy of proteins, the spectra in the ultraviolet region can be divided into near and far UV regions. While CD signals in the short-wave UV region of 170–260 nm (far UV region) are due to peptide binding, aromatic amino acids and disulfide bridges absorb in the long-wave UV region (near UV region) of 250–300 nm [48,72,73]. Thus, near-UV CD spectroscopy can provide information about the tertiary structure of proteins, while far-UV CD spectroscopy allows conclusions about the secondary structure of proteins or is suitable for characterizing the secondary structure of proteins [49,77,78,79,80,81,82,83,84].

**Figure 10:**
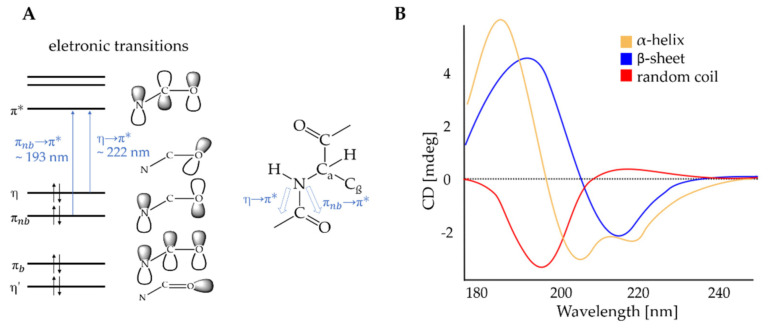
(**A**) Energy level diagram with electronic transitions of a peptide bond in the far UV region. Bonding, non-bonding, and antibonding p-orbitals ($\pi_{b},\;\pi_{nb}\;\mathrm{and}\;\pi^{*}$) and two free electron pairs at the oxygen $\left(\eta \;\mathrm{and}\;\eta \prime \right)$ are shown [75,76]. (**B**) Characteristic CD spectra in the far UV region of “pure” α-helix, β-sheet, and disordered structures, redrawn from [85].

Far UV CD spectroscopy was performed to evaluate the impact of conjugation of BITC on the conformational changes in the secondary structure of α-LA. The CD spectra of all samples in Figure 3 showed positive maxima at 190 nm and negative bands at approximately 208 and 220 nm. The CD bands in Figure 3 were attributed to electronic transitions of a peptide bond in the far UV region [75]. In this spectral region, the amides within the secondary structure components of a protein strongly absorb circularly polarized light and exhibit different numbers of electronic transitions for a given wavelength. Therefore, the CD spectrum of proteins in the far UV region is dominated by the absorption of the peptide bonds, and the absorption is dependent on the orientation and environment of the amide bond [85]. The peptide bond absorption is due to two electronic transitions: a strongly pronounced CD band of the $\pi \to \pi^{*}$ transition at 190 nm and a broader but weaker CD band of the des $\eta \to \pi^{*}$ transition between 210 and 220 nm (Figure 10A) [48,72,73,75,76,85,86,87]. In Figure 10B, the characteristic CD spectra of three secondary structures are shown for comparison with the experimentally obtained CD spectra (Figure 3). The CD spectrum of a “pure” α-helical structure shows two negative bands at 208 nm (*π* → π*) and 222 nm (*n* → π*) and a positive maximum at approximately 190 nm (*π* → π*) (orange) [87]. In “pure” β-sheet structures, a negative signal is observed at near 215 nm (*n* → π*) and a positive signal at about 196 nm (*π* → π*) [87]. The CD spectrum of the disordered structures stands out clearly. Here, a positive CD band at 212 nm and a negative one at 195 nm are characteristic [75,85].

For quantification of the secondary structural elements of a protein sample, the far-UV CD spectrum obtained is understood as the empirical sum of the fractional multiples of the characteristic spectra for each type of secondary structure type [86,88,89,90]. For quantitative analysis of the CD spectrum of a protein and estimation of the proportions of secondary structures, the experimentally obtained far-UV CD spectrum of a protein is understood as a linear combination of the three most abundant secondary structures (α-helix, β-sheet, and random coil) at a given wavelength. In reality, combinations or superpositions of several CD spectra of the respective secondary structure types are obtained. As there is a correlation between the far-UV CD spectra and the secondary structure of the proteins, CD spectroscopy can be used to determine the proportions of secondary structures and to study changes in secondary structure [86,88,89,90].

The far-UV spectra of the native and unmodified α-LA (Figure 3, black and red curves) showed a positive maximum at 190 nm and two negative bands at approximately 208 and 220 nm, which is characteristic of an α + β class protein [60,91]. In agreement with the results of other studies, quantitative determination of the amount of secondary structures confirmed that native α-LA consists of 30% α-helix, 12% β-sheet, and 58% of regions with low structural complexity (Table 2) [36,92,93]. It has been described in the literature that the secondary structure of native α-LA consists of 26% α-helices, 14% β-sheets, and 60% disordered structures [36], and the tertiary structure consists of two domains [36,37,94]. Although a large cleft separates the large α-domain from the small β-domain, they are held together by an ionic calcium bond and the four disulfide bridges [35,36,95]. Three pH-stable α-helices (H1 (5–11), H2 (23–34) and H3 (86–98)) and a pH-dependent α-helix (H4 (105–110) and two smaller 310 helices: (h1 (18–20) and h3 (115–118)) build the large α-domain, with the flexible loop region at positions 105–110 adopting a helical structure for pH values between 6.5 and 8 [96]. The ß-domain consists of three antiparallel ß-sheets (S1 (41–44), S2 (47–50), S3 (55–56)), a short 310 helix (h1b (18–20), h2 (77–80), h3c (115–118)), and loops and disordered structures [32,37,95,97].

The unmodified α-LA control also showed comparable amounts of secondary structural elements, suggesting that neither the synthesis nor the work-up conditions have an effect on the structural properties of the protein. While the values of the molar ellipticity of the positive band and the negative bands between the BITC-α-LA derivative “low” and the control samples showed no significant differences (Figure 3, dark green curve), the quantitative determination of the secondary structures of the BITC-α-LA derivative “low” showed a slight decrease in the α-helical structure to 24% and an increase in the β-sheet content to 19%. The amount of lower complexity secondary structures remained almost constant. As a result of increasing the concentration of BITC used to synthesize the BITC-α-LA derivatives “medium” and “high,” derivatization with BITC caused a decrease in band intensity at all wavelengths (Figure 3, light green and yellow curves). Increasing derivatization with BITC caused a further decrease in the α-helix content to 20 and 17%, with a concomitant increase in the ß-sheet structure content to 25 and 28%. The content of disordered structures remained unchanged. The results showed an influence on the secondary structure as a consequence of the BITC conjugation. As the degree of derivatization increased, a significant perturbation or successive change in the secondary structure of α-LA was documented. In summary, with increasing input concentration of BITC, a steady loss of α-helical fractions with a concomitant increase in the fractions of β-sheets was observed. Comparable results were recently obtained in a study investigating the conjugation of α-LA with polysaccharides [98]. The spectra of a pure and a conjugated protein were recorded by far UV-CD spectroscopy. Depending on the polysaccharides used, different degrees of reductions in α-helix fractions were observed [98]. Likewise, protein modification with, for example, polyphenols, oligosaccharides, and allicin showed a change in the secondary structure or conformation of proteins [99,100,101]. Rade-Kukic et al. (2011) also observed a change in secondary and tertiary structure depending on the amount of AITC bound to β-LG [1]. According to Kelly et al. (2005), an increase in molar ellipticities characteristic of α-helices may imply higher structural compactness. Conversely, the decrease in molar ellipticity of BITC-treated proteins would indicate less dense structures [73]. Similar findings were provided by the work of Das et al. (2014), who modified lysozyme, a structurally similar protein to α-LA, with different concentrations of ellipticin and then investigated the secondary structure using far-UV CD spectroscopy. By decreasing the band intensity between 208 and 230 nm, it was concluded that the typical α-helical structure of the protein was destabilized and unfolded as a result of the conjugation of ellipticin [102]. Chemical modification such as acetylations and sulfamidations as well as reactions with other secondary plant compounds such as polyphenols also resulted in similar restructuring and disruption of the secondary structure of the proteins, as reported by Gerbanowski et al. (1999), Schwenke et al. (2000), and Rawel et al. (2002, 2003) [60,91,103,104]. Sun et al. (2018) demonstrated that the addition of tetracycline hydrochloride (TCH) caused a loosening of the protein structure of lactoferrin. The CD spectra showed a decrease in negative band intensity as a result of the increasing degree of derivatization with TCH. They explained the reduction by breaking of hydrogen bonds due to TCH conjugation of lactoferrin and hypothesized that the altered secondary structure might also affect physiological functions [51].

It should be mentioned that the analysis of the amounts of β-sheets is complicated because this secondary structure produces only a relatively weak signal and cannot always be clearly differentiated from the signals of unfolded or disordered regions. Although it is not possible to obtain more detailed information about the exact secondary structure of proteins using CD spectroscopy, the additional value of the method is the ability to indicate denaturation or a structural change resulting from chemical modification and the ability to estimate the amounts of secondary structures, including α-helix, β-sheet, and disordered structures. For such studies, comparison with a CD spectrum of the native protein is indispensable [48,105]. More sensitive methods that can decipher smaller changes at the molecular level include fluorescence techniques [48,105].

### 3.3. Influence of BITC Conjugation on Temperature-Induced Conformational Changes of α-LA

The results of the CD experiments of the untreated α-LA control (Figure 4a) showed a decrease in the molar ellipticities of the double minimum and an increase in the molar ellipticities of the maximum at 190 nm in the temperature range from 30 to 50 °C, which could be indicative of higher structural compactness in the secondary structure according to Kelly et al. (2005) [73]. By further increasing the temperature (60–80 °C), a decrease in the minimum at 208 nm and the maximum at 190 nm, as well as a simultaneous increase in the minimum at 222 nm, were observed. These observations are indicative of a loss or a change of the secondary structure due to the increasing temperature.

Similar results were documented in the work of Lam et al. (2015). Although their focus was on the thermal pretreatment of the protein α-LA and subsequent study of the effect using CD spectroscopy, certain parallels are noted. They were able to observe a decreased mean ellipticity curve when the temperature was increased from 25 to 65 °C, suggesting increased order in the secondary structures of α-LA. By further increasing the temperature to 95 °C, a loss of secondary structure was assumed, which was reflected in an increase in the mean ellipticity curve in the wavelength range from 190 to 230 nm. They hypothesized that the protein underwent increased conformational entropy at temperatures up to 65 °C, which would result in the higher ordering of the polypeptide chains, whereas denaturing processes predominated at temperatures of 95 °C. Due to the fact that hydrogen bonds are broken at these temperatures, loss of secondary structure may consequently occur [28]. Another study confirmed that α-LA denatures irreversibly at temperatures above 90 °C [106].

For quantification of the α-helical portion of a protein, for example, CD data of molar ellipticity at 222 nm can be used, where the α-helical structure has a characteristic minimum ellipticity [85,107]. When comparing signals at a wavelength of 222 nm, differences between a folded and an unfolded protein can be large. However, an analogous method for estimating β-sheet or random coil structures does not exist [90,108]. For the derivatives (Figure 4b–d), an increase in the molar ellipticity of the minimum at 222 nm and a decrease in the maximum at 190 nm could already be noted at temperatures above 40 °C, suggesting a decrease in the α-helical fraction due to the temperature increase. In the α-LA control, the described effects could be observed only above a temperature of 60 °C.

Although the secondary structure appears relatively stable, it can be hypothesized that in addition to a dominant loss of the α-helical structure, an increase in the fractions of random coil or extended structures also affects the minimum at 209 nm. Thus, the decrease in the molar ellipticity of the minimum at 209 nm at temperatures above 50 °C for the α-LA control and for the BITC-α-LA derivative “small” could indicate a change in secondary structure. Considering Figure 10B, which shows the characteristic CD spectra of pure secondary structures, this relationship can be better visualized. While the fraction of the yellow curve (characteristic CD spectrum for proteins with pure α -helical structure) is decreased, the fraction of the blue (characteristic CD spectrum for proteins with pure β-sheet structure) and red (characteristic CD spectrum for proteins with random coil structure) curves can be increased. Presumably, this state is similar to the state that the BITC-α-LA derivatives “medium” and “high” already have with less thermal energy already at room temperature. The BITC-α-LA derivatives “medium” and “high” have overall lower band intensities and thus appear to be less “stably” folded than the α-LA control and the BITC-α-LA derivative “low”.

It can be summarized that with an increasing degree of derivatization, the differences of the molar ellipticities become increasingly smaller as a function of temperature. As already found out in Section 2.2, a change in the secondary structure fractions is evident with increasing BITC modification, although this difference between the samples could be increasingly lost at higher temperatures due to partial denaturation.

### 3.4. Influence of BITC Conjugation on Surface Hydrophobicity of α-LA

A commonly used extrinsic fluorescent dye is ANS, which is from the sulfonic acid group and is used to study unfolding or folding intermediates, detect protein aggregates, characterize changes in protein conformations, and measure surface hydrophobicity. Interaction of ANS with proteins is possible in two ways. On the one hand, there can be electrostatic interactions between the sulfonate group of ANS with positively charged side chains of the protein, and on the other hand, the interaction of ANS can occur via hydrophobic interactions with the hydrophobic regions on the surface of proteins [1,52,53,54,55,56,57,58]. While ANS does not fluoresce in aqueous polar solutions, the dye shows a blue shift of the fluorescence emission maximum and an increase in fluorescence intensity in apolar organic (hydrophobic) solutions or when bound to, for example, proteins [52,56,58,109,110,111,112].

The results in Figure 5 showed that unmodified α-LA exhibited low ANS fluorescence emission, suggesting that ANS molecules interacted with hydrophobic regions on the surface of the protein. Globular, water-soluble proteins in the native state, such as α-LA, are thought to have low affinity or accessibility for ANS molecules due to the orientation of the hydrophobic protein side chains into the interior of the molecule. Although the hydrophobic core of most protein is typically shielded from the organic environment by a rigid tertiary structure, isolated hydrophobic groups may occur on the protein surface or be exposed in crevices so that even native globular proteins may have a few hydrophobic binding sites on the protein surface for ANS molecules and produce low fluorescence intensity [109,110,113]. This assumption is supported by previous results showing that native α-LA can bind up to five molecules of an ANS derivative [35,114]. In work described by Singh et al. (2006), it was added that the binding of ANS to the native state of α-LA was not very strong due to the lack of hydrophobic interactions [114].

Thus, while in the native, untreated state of α-LA, the hydrophobic side chains are mainly concentrated in the protein interior and are of limited availability to the ANS molecules, and an increasing ANS fluorescence intensity was documented with increasing degree of derivatization, suggesting that BITC conjugation exposed increased hydrophobic regions on the surface of α-LA and allowed them to interact with the ANS molecules. The resulting increase in binding affinity to ANS explained the increase in ANS fluorescence as a function of the concentration of BITC used. In summary, BITC conjugation resulted in a change of the hydrophobic character, more specifically, an increase in the surface hydrophobicity of α-LA [9].

Previous research regarding the change in surface hydrophobicity of other proteins showed similar effects after chemical modification. For example, a correlation between the conformational change and the increase in surface hydrophobicity of concanavalin A (ConA) as a result of the conjugation of sodiumdodecylsulfate (SDS) was documented [115]. Additionally, modifications of proteins, such as BSA, casein, and whey protein isolates, with citric acid or acyl groups (acylations) led to an increase in surface hydrophobicity with increasing degree of derivatization, which was attributed to a change in protein conformation and subsequent exposure of hydrophobic groups to the protein surface [116,117]. The relationship between the exposure of hydrophobic groups due to denaturation of proteins and the increase in surface hydrophobicity was described as early as 1980 by Kato et al. [118].

Wilde et al. (2016) studied the interaction of β-LG with allicin and diallyl disulfide using RP-HPLC and far UV-CD spectroscopy to detect changes in surface hydrophobicity and secondary structure. They described the conjugation of the ligands and the resulting change in the secondary structure or loosening of the globular protein structure as the cause of the increased surface hydrophobicity of the protein [99]. In a follow-up study, Keppler et al. (2017) found that under neutral conditions, ANS binding was increased as a result of AITC conjugation to whey protein isolate (WPI). The increase in hydrophobic surface area was probably caused by changes in the conformation of WPI as a result of conjugation of the hydrophobic ligand AITC [9]. In another study, AITC conjugation to the whey protein β-LG was investigated [1]. The results of ANS measurements initially showed an increase in fluorescence intensity under neutral conditions, whereas there was a decrease in fluorescence emission as the degree of derivatization increased. The initial increase in ANS fluorescence was consistent with RP-HPLC results, which also showed an increase in hydrophobicity. The subsequent decrease in ANS fluorescence intensity due to increasing AITC modification was attributed to the change in protein conformation confirmed by CD experiments. The authors concluded that the increased surface hydrophobicity resulted from the AITC-induced conformational changes. Furthermore, improved emulsifying and foaming properties of the protein were obtained as a result of AITC conjugation to β-LG [1]. Similar effects of chemical modification on emulsifying and foaming properties of WPI were reported by Li et al. (2018) [116].

While conjugation with phenols on BSA resulted in a decrease in hydrophobic surface area and improved solubility [91], phenol adducts with myoglobin showed opposite effects. The myoglobin–phenol conjugates showed increased hydrophobicity and thus decreased solubility [119,120]. Another study confirmed the correlation between increased surface hydrophobicity and decreased solubility by ANS fluorescence measurements [59].

Considering that the electrophilic ITC preferentially react with amino and thiol groups of the protein side chains, it is reasonable to assume that as a consequence of the introduction of the hydrophobic ligand BITC or by blocking the free amino and thiol groups, a decrease in polarity and a loss of charge cause the increase in surface hydrophobicity. In addition, the ITC-induced change in secondary structure favors the exposure of hydrophobic regions on the protein surface [1].

### 3.5. Influence of BITC Conjugation on the Hydrodynamic Radius of α-LA

Dynamic light scattering (DLS) is an analytical method for determining the size and size distribution and monitoring the aggregation behavior and ligand binding of proteins and other biomolecules, usually in the nanometer to submicrometer range [121,122,123]. The method measures the Brownian molecular motion of particles or molecules dissolved or dispersed in a liquid and uses the information to calculate the hydrodynamic radius [121,122,123,124,125].

Determination of the mean hydrodynamic radius of the unmodified α-LA control yielded a value of 1.76 ± 0.24 nm, which is comparable to previously published values [95,126,127]. For example, Delavari et al. (2015) were able to determine a hydrodynamic diameter of 3.6 nm for α-LA [95]. As a result of the conjugation of BITC to α-LA, a significant increase in the hydrodynamic radius to a maximum of 89.37 ± 14.86 nm was observed regardless of the concentration of BITC used. Furthermore, multiple signals and broader peaks were observed, indicating inhomogeneous and highly polydisperse protein solutions.

Similar effects on the hydrodynamic radius of proteins as a result of interaction with ligands were documented by Delavari et al. (2015) and Abasi et al. (2014) [95,128]. Thus, the interaction of α-LA with vitamin D3 showed an increase in hydrodynamic diameter from 3.6 to 125 nm. Furthermore, they found an altered secondary structure and increased surface hydrophobicity as a result of the interaction [95]. Abasi et al. (2014) reported a nanoparticle consisting of vitamin D3 and homogenized whey protein isolate with similar size [95,128]. Presumably, the increase in particle size is due to the encapsulation of vitamin D3 to the α-LA [128] or hydrophobic intramolecular interactions between the protein molecules [129]. It has been suggested that upon binding of vitamin D3 to α-LA, hydrophobic side chains are exposed, which may allow hydrophobic interactions between protein molecules, leading to larger complexes [129]. A further study showed that when oleic acid was used as a hydrophobic ligand, the α-LA derivatives were larger than those of other proteins [126].

It should be noted that in DLS, an intensity-weighted hydrodynamic radius is determined, and this is strongly dominated by larger particles/molecules at the expense of smaller particles/molecules [1,130]. That is, due to the fact that the intensity of the scattered light increases proportionally to the sixth power of the diameter, the scattered light from a small population of large particles/molecules overlaps the scattered light from smaller particles to such an extent that they can no longer be detected, leading to biased radius distributions [131]. For a complementary quantitative analysis of particle sizes in polydisperse solutions, alternative methods, such as size exclusion chromatography, should be used [130].

### 3.6. Influence of BITC Conjugation on Tryptic Digestion of α-LA

As trypsin preferentially cleaves after the amino acids lysine and asparagine [132] and ITC has been shown to react with e-amino groups of lysine side chains several times [43,45,67,69], it is reasonable to assume that BITC modification of the amino group of the amino acid lysine could lead to masking of trypsin cleavage sites/steric hindrance of tryptic hydrolysis [7,17,42,132]. This assumption can be confirmed by the present results, which showed that more elongated modified peptide sequences were detected with increasing derivatization. Furthermore, this assumption was supported by the fact that the tryptic hydrolysis of the BITC-modified α-LA derivatives yielded a higher amount of peptides compared to that of the native α-LA control sample, again emphasizing that as a result of the conjugation of BITC to the amino groups of lysine, the protein is protected from subsequent tryptic hydrolysis [7,65]. The present findings were in agreement with the results of previous studies, which also showed that tryptic hydrolysis was inhibited for ITC-modified β-lactoglobulin [7], egg white proteins, myoglobin, legumin [133], and glycosylated proteins [134,135].

Moreover, the results of mass spectrometry analysis showed that the BITC-modified peptides eluted at later retention times, which was consistent with the experimentally obtained ANS fluorescence results, indicating higher hydrophobicity. Similar results were shown in the work of Wilde et al. (2016), who also linked an increase in retention time to an increase in hydrophobicity as a result of the modification of β-LG by allicin and diallyl disulfide [99].

In agreement with previous results, it was repeatedly shown that a higher degree of derivatization could be achieved with increasing input concentration of BITC, resulting in successive blocking of several tryptic cleavage sites of α-LA and consequently yielding longer peptides [7].

It is conceivable that via the classical CID fragmentation method, a clear localization of the modification is hardly possible due to the complete cleavage of the BITC group. In contrast to CID, newer fragmentation techniques are based on a destabilization of the peptide ions via a reaction with electrons (electron-capture dissociation, ECD) or radical anions (electron-transfer dissociation, ETD) [136]. ECD and ETD are particularly used to characterize labile protein modifications, preferentially generating c- and z-fragment ions. A key advantage of these methods is that modifications that are unstable under collision-induced fragmentation remain intact, allowing unambiguous localization. Thus, labile histidine and lysine modifications/phosphorylations can be identified [137].

## 4. Materials and Methods

### 4.1. Materials

1,4-Dioxane, acetonitrile, ethanol, disodium hydrogen phosphate, hydrochloric acid (32%), isoleucine, sodium dihydrogen phosphate, sodium hydrogen carbonate, and dialysis membranes (regenerated cellulose, molecular weight cutoff < 3.5 kDa) were obtained from Carl Roth GmbH & Co. KG, Karlsruhe, Germany. Bovine α-lactalbumin (α-LA) as model protein, benzyl isothiocyanate (98%), dithiothreitol (DTT), *n*-acetyl-l-cysteine, o-phthalaldehyde, 8-Anilino-1-naphthalenesulfonic acid ammonium salt, and trypsin from porcine pancreas were purchased from Sigma-Aldrich GmbH, Steinheim, Germany. Formic acid and urea were purchased from Merck KGaA, Darmstadt, Germany. C_18_ solid-phase extraction cartridges (1 mL, 100 mg) were purchased from Macherey-Nagel GmbH & Co. KG, Düren, Germany. All solvents were of HPLC grade; otherwise, ACS grade was used. Water was double-distilled (ddH_2_O).

### 4.2. Methods

#### 4.2.1. Preparation of ITC-α-LA Conjugates

The preparation of the ITC-protein conjugates was carried out according to the instructions of Spöttel et al. [47]. Briefly, for the synthesis of ITC-protein conjugates, α-LA protein was first dissolved in water (0.714 mM) and then mixed with three to six different concentrations of BITC ranging from 0 to 113 mM (Table 7). For this purpose, the appropriate amount of BITC was dissolved in 1,4-dioxane. After the incubation for 20 h at 37 °C, the reaction solution was dialyzed overnight and subsequently lyophilized for removing as much as residual BITC. To prevent cold denaturation, for lyophilization, the samples were first frozen in liquid nitrogen and then transferred to a laboratory freeze dryer (Christ RVC 2−25 CDplus, Martin Christ Gefriertrocknungsanlagen GmbH, Osterode am Harz, Germany). In order to make a comparison between BITC-treated and untreated α-LA and to be able to exclude any influence of the synthesis and work-up conditions, control samples were prepared and treated in the same way as the modifications except that no BITC was added. The freeze-dried samples were stored at −20 °C until analysis. Depending on the subsequent analysis, the freeze-dried sample was dissolved in a suitable solvent.

#### 4.2.2. Determination of Free Amino Groups Using *O*-Phthaldialdehyde

A commonly used reagent for the determination of free α- and ε- amino groups is *ortho*-phthaldialdehyde (OPA), which reacts with primary amino groups in the presence of a thiol compound such as *N*-acetyl-l-cysteine (NAC) to form a 1-alkylthio-2-alkyl-substituted isoindole. The fluorescent isoindole can absorb light at a wavelength of 340 nm and has been quantified spectrophotometrically [1,138,139,140,141,142,143].

For the quantitative determination of free α- and ε-amino groups before and after conjugation of BITC, the method using OPA was performed [144]. For this purpose, the protocol provided by the Interchim Deutschland GmbH (Mannheim, Germany) was used as a basis for the measurement of free amino groups in proteins, which was slightly adapted [145]. Major differences were that instead of α-acetyl-lysine, isoleucine was used as a standard, and instead of mercaptoethanol, *N*-acetyl-l-cysteine was used as a thiol component. The isoleucine used contains one amine per molecule, allowing a ratio of labeling to be determined.

Briefly, for the quantitative determination of free amino groups of unmodified and BITC-modified α-LA, six different modifications and a control sample were prepared as described previously (Table 7, Section 4.2.1). The lyophilized samples were dissolved in 2 mL carbonate buffer (50 mM, pH 10) and each diluted 1:5 (71.4 µM). To prepare the OPA reagent, 5 mg OPA and 11.7 mg *N*-acetyl-l-cysteine (NAC) were dissolved in 100 µL ethanol followed by 10 mL carbonate buffer (50 mM, pH 10). The OPA reagent was protected from direct light and used within two hours. For the external calibration series, a 10 mM isoleucine standard solution was prepared in carbonate buffer followed by a serial dilution of 10–522 µM of the standard solution in carbonate buffer. The preparation of the calibration series was performed with seven calibration points. A total of 100 µL of OPA reagent was added to 100 µL of each of the calibration solutions and the protein solutions to be examined and incubated for two minutes at 23 °C in a 96-well microtiter plate. Transparent plates were used for the measurement. Subsequently, the absorbance was determined photometrically at a wavelength of 340 nm, using a Synergy^TM^ HT (BioTek Instruments Inc., Vermont, VT, USA). A comparison was made between the calculated free amino groups of the control sample and those of the BITC-modified derivatives. For this purpose, the concentration of free amino groups of the control sample was normalized to 100%. The assay of accessible amino groups was performed in triplicate.

#### 4.2.3. Investigation of Secondary Structure by Far-UV CD Spectroscopy

CD spectroscopy was used to evaluate the influence of BITC conjugation on the secondary structure of α-LA. In order to estimate and compare the composition of the secondary structures of the untreated α-LA control and the BITC-α-LA derivatives “low,” “medium,” and “high,” CD experiments were performed using a Jasco J-815 spectrometer (Jasco Inc., Mary’s Court, MD, USA).

After lyophilization, the samples under investigation were dissolved in 1 mL of 0.01 M sodium phosphate buffer (pH 8) and diluted to a final protein concentration of approximately 16–18 µM. When selecting the buffer, it is important to ensure that the buffer used has the lowest possible self-absorption in the short wavelength region to minimize signal interference. In addition, a native protein control was measured to exclude any impact of synthesis and purification on the protein structure. For this purpose, 1 mg of α-LA was weighed, dissolved in the sodium phosphate buffer, and diluted.

The CD experiments were performed under permanent nitrogen flow to protect the optical components of the spectropolarimeter from ozone gas. Ozone gas can be formed from atmospheric oxygen under UV irradiation, which can attack the mirror surfaces and consequently reduce the reflectivity and longevity of the mirrors and reduce the efficiency in focusing the light through the monochromator. In addition, molecular oxygen absorbs below 195 nm, but since this spectral region is particularly important for estimating the secondary structure of proteins, the lower the wavelength during the measurement, the greater the flux rate of nitrogen must be to minimize oxygen absorption.

Measurements were performed in the far-UV range between 185 and 260 nm in a quartz cell of 1 mm slice thickness, a step size of 0.1 nm per data point, and a scanning speed of 100 nm/min. The temperature was set to 20 °C for all measurements using a Peltier element (Jasco Inc., Mary’s Court, MD, USA). For each sample, 15 spectra were recorded and averaged. Then, the averaged ellipticity of the buffer solution was subtracted from the CD data of all samples. The measured ellipticities were scaled and expressed as molar ellipticity (MME) in deg cm^2^ dmol^−1^:

$$\left[\theta \right]=\frac{\theta \cdot M}{d\cdot c\cdot 100,000}$$

where θ is the ellipticity in mdeg, *M* is the molecular weight of the protein in g/mol, *d* is the path length of the cuvette in cm, and *c* is the concentration of the protein in g/mL.

The experimentally obtained spectra provided a fingerprint of the secondary structure composition of untreated and BITC-treated α-LA.

Online services can be used to facilitate the analysis of proteins. To evaluate the data, reference spectra of proteins with known 3D structures listed in an available database (the protein circular dichroism data bank, or PCDDB) are used. In addition, the CD spectroscopic data were analyzed using the DichroWeb online server (http://dichroweb.cryst.bbk.ac.uk/html/links.shtml; accessed on 10 October 2021). DichroWeb provides several algorithms to deconvolute CD spectra. These include, for example, the K2d method [146], which has integrated protein reference data [147]. The K2D method of the Dichroweb server was used to deconvolute the CD data and determine the relative secondary structure composition of each sample. Thus, based on a far-UV CD spectrum of a protein, the percentages of the respective secondary structures were calculated [75].

#### 4.2.4. Investigation of Temperature-Induced Conformational Changes Using Far UV CD Spectroscopy

For the analysis of the temperature-induced change in the secondary structures of the untreated α-LA control and the BITC-α-LA derivatives “low,” “medium,” and “high,” the samples were prepared, measured, and analyzed according to Section 4.2.3. Major deviation in the performance was that the CD spectra were recorded using the Peltier element (Jasco, 21601 MD, USA) in a temperature range of 30–80 °C with an increment of 2 °C/min. To investigate the temperature stability of the protein solutions, the temperature was increased by 2 °C per minute to adjust and stabilize the temperature for the next measurement.

#### 4.2.5. Investigation of Surface Hydrophobicity Using ANS Fluorescence

To measure the surface hydrophobicity of untreated and BITC-treated α-LA, 8-anilinonaphthalene-1-sulfonic acid was used as hydrophobic fluorescent probe that interacts with hydrophobic/nonpolar regions on the protein surface and generates a fluorescent signal [56,110,118,148]. The measurements were performed following the work of Kato et al. [118] and Lam et al. [149].

BITC-treated and untreated protein samples were prepared as previously described. After lyophilization, the freeze-dried samples were dissolved in 2 mL of phosphate buffer (0.01 M, pH 8) to give a protein concentration of 0.035 mM. To prepare the ANS reagent, 29.0 mg of ANS ammonium salt was dissolved in 10 mL of demineralized water (9.17 mM). After aliquoting the protein solutions into three parts of 0.4 mL each, 77.9 µL of ANS reagent was added to each part, resulting in a protein/ANS concentration ratio of 1/50. The protein solutions were incubated for five minutes in the absence of light. Subsequently, the measurement was performed using a 384-well black corning flat bottom plate on the SpectraMax^®^ M3 fluorescence microplate reader from Moleculare Devices LLC (San Jose, CA, USA). For this purpose, 80 µL of each sample solution was pipetted three times into the microplate, and then the fluorescence was measured at an excitation wavelength of 390 nm in the wavelength range of 430–600 nm. The surface hydrophobicity was determined in triplicate for each sample.

#### 4.2.6. Determination of the Hydrodynamic Radius Using Dynamic Light Scattering

Dynamic light scattering (DLS) can be used to obtain information on the aggregation behavior, hydrodynamic radius, and monodispersity of BITC-modified and unmodified α-LA (derivatives). DLS measurements were performed using a SpectroSize300 instrument with an implemented 660 nm wavelength diode laser (Xtal-Concepts GmbH, Germany). After synthesis, the unmodified α-LA control sample and the BITC- α-LA derivatives “low,” “medium,” “high,” and “very high” were centrifuged at 16,000× g for 10 min and then transferred to a quartz cuvette without air bubbles. The measurement was carried out at a temperature of 20 °C and a scattering angle of 90° (viscosity of the sample: 2.18 cP; refractive index: 1.33). For each sample, 20 DLS measurements for 20 s per measurement were performed. The hydrodynamic radius was calculated as an average value from the 20 scans. The particle size distribution was calculated using the CONTIN algorithm, taking into account the viscosity of the medium. The diffusion coefficient D was determined based on an autocorrelation analysis of the scattered light intensity governed by translational particle diffusion, and the average hydrodynamic radius was then calculated using the Stokes–Einstein equation:

$$R_{H}=\frac{kT}{6\pi \eta D}$$

where *R_H_* is the hydrodynamic radius (m), *k* is the Boltzmann constant [1.380642 × 10^−23^ JK^−1^], *T* is the absolute temperature (K), *η* is the viscosity of the solution [kg m^−1^ s^−1^], and *D* is the translational diffusion coefficient [m^2^ s^−1^] [150].

#### 4.2.7. Tryptic Hydrolysis of Unmodified and BITC-Modified α-LA

Tryptic hydrolysis of the freeze-dried BITC-treated and untreated protein samples was performed according to the instructions described by Spöttel et al. [47]. For this purpose, the freeze-dried samples were resolved in 50 µL of 6 M aqueous urea followed by addition of 100 mM DTT (in 100 mM sodium hydrogen carbonate) to cleave disulfide bridges. After incubation at 60 °C for 10 min, 425 µL 100 mM sodium hydrogen carbonate (in water) was added. Tryptic hydrolysis was performed by adding the trypsin solution (1 mg/mL in 0.1 mM HCl) to the protein solution at a ratio of 1:100 for 16 h at 37 °C. Subsequently, the enzymatic hydrolysis was terminated by the addition of 0.2% formic acid. Solid-phase extraction was used to purify the digested samples. Therefore, the chromatography material was first conditioned and equilibrated with 60% ACN (in water) and 0.2% formic acid (in water). Subsequently, the samples were applied, rinsed with 0.2% formic acid (in water), and then eluted with 60% ACN (in water). Finally, the samples were concentrated with gaseous nitrogen and dissolved in 0.2% formic acid (in water) for further analysis.

#### 4.2.8. LC-ESI-MS/MS of Unmodified and BITC-Modified α-LA

Chromatographic separation of tryptic untreated and BITC-modified protein hydrolysates was performed on a reversed-phase HPLC column (Nucleodur, 5 µm C8 100 Å, 150 × 2 mm) from Macherey-Nagel GmbH & Co. KG (Düren, Germany) and a Dionex UltiMate™ 3000 UHPLC system (Thermo Fisher Scientific Inc., Waltham, MA, USA).

The mobile phase consisted of water (A) and acetonitrile (B), each with 0.1% formic acid. Gradient elution was performed in several steps, starting at 97% A for 5 min, decreasing linearly to 80% A in 10 min, and kept constant for 5 min. Subsequently, there was a further reduction in mobile phase A to 70% A in 5 min, followed by a plateau for 5 min and a final decrease to 30% A in 10 min, which was held constant for 6 min. The gradient was returned to 97% A in 4 min, followed by a 15 min re-equilibration.

The injection volume was 10 µL at a flow rate of 200 µL/min for all samples. The LC-MS system was controlled by HyStar 3.2 (Bruker Daltonik GmbH, Bremen, Germany).

Detection of the previously chromatographically separated tryptic untreated and BITC-modified protein hydrolysates was performed using an ESI-MS ion trap mass analyzer in positive ion mode (amazon speed ETD, Bruker Daltonik GmbH, Bremen, Germany) with the following settings analyzed: ion spray voltage: 4.5 kV; ion source heating: 350 °C; source gas: 55 psi. Using the UniProtKB database (http://www.uniprot.org/; accessed on 20 September 2021) and the SIB Bioinformatics Resource Portal ExPASy (https://www.expasy.org/; accessed on 20 September 2021), the experimentally obtained data could be compared with a theoretical digest of α-LA so that the obtained signals could be assigned and the resulting peptides could be identified.

## 5. Conclusions

In summary, it was shown that with increasing input concentration of BITC, an increasing degree of derivatization could be achieved, which was accompanied by a successive change in the secondary structure of α-LA (loss of α-helix moieties, with a concomitant increase in β-sheet moieties). The restructuring was related to the increased surface hydrophobicity because the change of the secondary structure was assumed to expose more hydrophobic regions on the protein surface. Accordingly, the change in protein structure resulting from BITC conjugation may have exposed hydrophobic regions (increase in surface hydrophobicity). This further enables hydrophobic interactions between multiple protein molecules, leading to larger α-LA complexes/aggregates (increase in hydrodynamic radius) [129,151], which could potentially be promoted via surface-exposed ß-sheet structure. This assumption could explain the increase in hydrodynamic radius due to BITC modification. In addition, the mass spectrometric analysis revealed that as a result of BITC modification, the tryptic digestibility of α-LA was decreased, which was attributed to steric blocking of the tryptic cleavage sites and the two modified amino acids that were located at the lysine side chains (K32 and K113) in the amino acid sequence of α-LA.

These studies are of particular importance in order to assess the influence of chemical modifications on the molecular structure of proteins since structural changes can alter the functional and biological properties of proteins [48,49,72,75,76]. Furthermore, the determination of freely available amino groups plays a crucial role with regard to the properties and functionality of proteins [1,152]. Knowledge of the influence of chemical modifications on protein structure and surface hydrophobicity allows conclusions to be drawn about solubility, aggregation, and physical stability [52,153], which are particularly important with regard to food processing and food safety [1,51].

## Figures and Tables

**Table 1:** Free amino group content of the unmodified α-LA and BITC-α-LA derivatives.

Sample	B_BITC/α-LA_	Free-NH_2_ Content (%)	SD (%)
α-LA control	0	100	-
BITC-α-LA derivative “minimal”	10	87.2	3.6
BITC-α-LA derivative “minor”	25	88.0	2.0
BITC-α-LA derivative “low”	50	79.3	2.1
BITC-α-LA derivative “medium”	500	61.8	17.5
BITC-α-LA derivative “high”	1000	59.9	15.4
BITC-α-LA derivative “very high”	1500	59.0	8.8

**Table 2:** Quantification of the secondary structural composition of the proteins at 20 °C and 80 °C.

Sample	Temperature (°C)	α-Helix (%)	β-Sheet (%)	Random Coil (%)
LA (native)	20	30	12	58
α-LA control	20	29	13	58
	80	15	15	70
BITC-α-LA-derivative “low”	20	24	19	57
	80	19	12	69
BITC-α-LA-derivative “medium”	20	20	25	56
	80	18	19	63
BITC-α-LA-derivative “high”	20	17	28	55
	80	16	26	58

**Table 3:** Averaged hydrodynamic radii (*R_H_*) along with standard deviations (SD) of α-LA control and BITC-α-LA derivatives (*n* = 20 scans).

Sample	No.	R_H_ (± SD) (nm)	No.	R_H_ (± SD) (nm)
α-LA control	I	1.8 (±0.2)		
BITC-α-LA-derivative “low”	I	2.4 (±0.5)	II	2.9 (±0.7)
	III	13.8 (±3.2)	IV	15.8 (±3.5)
	V	57.8 (±11.5)		
BITC-α-LA-derivative “medium”	I	83.8 (±18.3)	II	89.4 (±14.9)
BITC-α-LA-derivative “high”	I	2.0 (±0.3)	II	55.5 (±11.3)
	III	61.0 (±13.6)		
BITC-α-LA-derivative “very high”	I	51.6 (±11.7)	II	62.1 (±11.8)
	III	68.6 (±15.6)		

**Table 4:** Summary of all identified peptides after tryptic hydrolysis of the unmodified control sample (K) and BITC-α-LA derivatives. The numbering, the corresponding peptide mass, and the associated retention time are listed. Peptides that have been modified are marked with an “*”, while elongated peptides resulting from BITC modification were highlighted with “**”.

No.	Peptide Sequence	Mass (*m/z*)	Control	Low	Medium	High	Very High
1	EQLTK	618.35	10.7	10.9	11.2	11	11.2
1 *	EQLTK + BITC	767.56	-	29.7	29.8	30.5	30.3
2	CEVFR	653.31	19.1	-	-	-	-
3	ELK	389.24	4.5	4.5	4.6	4.5	4.6
3 *	ELK + BITC	538.45	-	-	-	-	29.3
3 **	ELKDLK + BITC	894.17	-	-	27.0	26.9	26.8
4	DLK	375.22	5.2	5.3	5.3	5.2	5.2
5	GYGGVSLPEWVCTTFHTSGYD-TQAIVQNNDST-EYGLFQINNK	4654.15	-	-	-	-	-
6	IWCK	549.29	18	-	-	-	-
7	DDQNPHSSNICNISCDK	1889.78	16.6	17.0	16.8	17	18.1
8	FLDDDLTDDIMCVK	1642.73	-	-	-	-	-
9	K	147.11	-	-	-	-	-
9 **	KILDK + BITC	765.61	-	23.4	23.3	23.2	23.3
10	ILDK	488.31	16.1	16.1	16.1	16.1	16.0
10 *	ILDK + BITC	637.52	-	-	-	32.5	32.7
11	VGINYWLAHK	1220.65	20.9	20.9	20.3	21.3	20.7
12	ALCSEK	650.32	16.4	15.9	15.8	15.7	16.2
12 **	ALCSEKLDQWLCEK + BITC	1815.1	-	-	-	-	31.8
13	LDQWLCEK	1034.50	20	21.2	23.5	24.5	23.2
13 *	LDQWLCEK + BITC	1183.71	-	34.1	33.8	34.4	33.4
14	L	132.10	-	-	-	-	-

**Table 5:** Mass of unmodified and modified *y*- and *b*-fragment ions of the sequence ELKDLK.

Sequence	No.	*b*-Ions	b*-Ions	*y*-Ions	y*-Ions	No.
E	1	130.05	279.26	745.45	894.66	6
L	2	243.13	392.34	616.40	765.61	5
K	3	371.23	520.44	503.32	652.53	4
D	4	486.26	635.47	375.22	524.43	3
L	5	599.34	748.55	260.20	409.41	2
K	6	727.43	876.64	247.11	296.32	1

**Table 6:** Mass of unmodified and modified *y*- and *b*-fragment ions of the sequence KILDK.

Sequence	No.	*b*-Ions	b*-Ions	*y*-Ions	y*-Ions	No.
K	1	129.10	278.31	616.40	765.61	5
I	2	242.19	391.40	488.31	637.52	4
L	3	355.27	504.48	375.22	524.43	3
D	4	470.30	619.51	262.14	411.35	2
K	5	598.39	747.60	147.11	296.32	1

**Table 7:** Summary of synthesis parameters to represent BITC protein conjugates and control sample.

Sample	c(α-LA) (mM)	c(BITC) (mM)	B_BITC/α-LA_
α-LA control	0.07	-	0
BITC-α-LA derivate ”minimal”	0.07	0.6	10
BITC-α-LA derivate “minor”	0.07	1.9	25
BITC-α-LA derivate ”low”	0.07	3.8	50
BITC-α-LA derivate “medium”	0.07	38	500
BITC-α-LA derivate “high”	0.07	75	1000
BITC-α-LA derivate “very high”	0.07	113	1500

## Data Availability

The data presented in this study are available on request from the authors.

## Sample Availability

Samples of the compounds are available from the authors.
